# Photosynthesis Response and Transcriptional Analysis: Dissecting the Role of SlHB8 in Regulating Drought Resistance in Tomato Plants

**DOI:** 10.3390/ijms242015498

**Published:** 2023-10-23

**Authors:** Yinghua Yang, Xinyue Zhang, Qiuxiang Zhong, Xiaojuan Liu, Hongling Guan, Riyuan Chen, Yanwei Hao, Xiaolong Yang

**Affiliations:** College of Horticulture, South China Agricultural University, Guangzhou 510642, China; yinghuayang@stu.scau.edu.cn (Y.Y.); xinyue_z2021@163.com (X.Z.); zqx15990173181@163.com (Q.Z.); liuxjjy628@stu.scau.edu.cn (X.L.); guanhl@scau.edu.cn (H.G.); rychen@scau.edu.cn (R.C.)

**Keywords:** aquaporin, chloroplast quality, photochemical reaction, stomatal aperture, transcriptional regulation

## Abstract

Deciphering drought resistance in crops is crucial for enhancing water productivity. Previous studies have highlighted the significant role of the transcription factor SlHB8 in regulating developmental processes in tomato plants but its involvement in drought resistance remains unclear. Here, gene overexpression (*SlHB8*-OE) and gene knockout (*slhb8*) tomato plants were utilized to study the role of SlHB8 in regulating drought resistance. Our findings showed that *slhb8* plants exhibited a robust resistant phenotype under drought stress conditions. The stomata of *slhb8* tomato leaves displayed significant closure, effectively mitigating the adverse effects of drought stress on photosynthetic efficiency. The *slhb8* plants exhibited a decrease in oxidative damage and a substantial increase in antioxidant enzyme activity. Moreover, *slhb8* effectively alleviated the degree of photoinhibition and chloroplast damage caused by drought stress. SlHB8 regulates the expression of numerous genes related to photosynthesis (such as *SlPSAN*, *SlPSAL*, *SlPSBP*, and *SlTIC62*) and stress signal transduction (such as *SlCIPK25*, *SlABA4*, and *SlJA2*) in response to drought stress. Additionally, *slhb8* plants exhibited enhanced water absorption capacity and upregulated expression of several aquaporin genes including *SlPIP1;3*, *SlPIP2;6*, *SlTIP3;1*, *SlNIP1;2*, and *SlXIP1;1*. Collectively, our findings suggest that SlHB8 plays a negative regulatory role in the drought resistance of tomato plants.

## 1. Introduction

Water is essential for plants’ survival but global agricultural production is being severely restricted by the increasing water scarcity caused by climate change [1]. Therefore, there is an urgent need to develop effective strategies to enhance the drought resistance of crops in order to achieve efficient water use and high yields. When plants sense water deficiency through their roots, specific stress signaling pathways are activated, leading to various physiological responses. These responses include the accumulation of stress-protective metabolites, activation of the antioxidant system to prevent cellular damage, increased water uptake by the roots, closure of stomata to reduce water loss, and adjustments in osmotic balance and ion homeostasis [2,3]. Understanding the mechanisms underlying drought resistance is crucial for developing solutions to improve water productivity without compromising crop yield.

Drought stress can quickly lead to the closure of stomata, which reduces water loss but also hinders the supply of carbon dioxide [4,5]. In severe cases, drought can also cause photoinhibition due to the excessive absorption of light energy. In addition to the regulation of stomatal dynamics, efficient photosynthetic electron transport is crucial for utilizing absorbed light energy in photochemical reactions and meeting metabolic demands. Short-term regulatory mechanisms, such as cyclic electron flow (CEF), nonphotochemical quenching (NPQ), and photosynthetic control, have evolved to maintain the balance of photochemical reactions during drought stress [6,7,8]. Prolonged drought stress can lead to the excessive accumulation of reactive oxygen species (ROS), which severely damages the photosynthetic apparatus and leads to chloroplast degradation [9,10,11].

In recent decades, numerous studies have revealed various mechanisms of drought resistance in plants, including the mediation of signal transduction by Ca^2+^, H_2_O_2_, and the phytohormone abscisic acid (ABA), as well as the regulation of key stress response transcription factors [1,2,3,4,10,12]. In our previous studies, we discovered that a class III homeodomain–leucine zipper (HD-Zip III) transcription factor named SlHB8 plays a crucial role in regulating leaf rolling, stem development, and pollen wall formation in tomato plants [13,14,15]. However, its role in stress resistance remains unclear. The mRNA of HD-Zip III family genes can be degraded by the evolutionarily conserved stress biomarker microRNA 166 [16]. Disruption of microRNA regulation sites leads to the upregulation of HD-Zip III genes and results in significant developmental phenotypes. Recently, it was found that, in tomato plants, the six HD-Zip III genes, including *SlHB8*, are negatively regulated by miR166 [17,18]. Furthermore, the involvement of HD-ZIP III family members in abiotic stress response has been extensively described in rice, cassava, barley, cucumber, and tea plants [19,20,21].

Tomato is a globally significant vegetable crop but its sustainable production is threatened by inadequate water supply during irrigation. Based on our previous research on the role of SlHB8 in controlling various developmental processes in tomato plants, as well as the aforementioned analysis, we hypothesized that SlHB8 may also be involved in regulating drought resistance in tomato. To investigate this, we conducted experiments using transgenic tomato plants with overexpressing of *SlHB8* and knocking out of gene lines through CRISPR/Cas9 technology. Our study was specifically focused on detecting the impact of SlHB8 on photosynthetic capacity, oxidative stress homeostasis, chloroplast quality maintenance, and transcriptional regulation of genes involving in drought resistance.

## 2. Results

### 2.1. Effects of SlHB8 on Stomatal Aperture and Photosynthetic Response of Tomato Leaves under Drought Stress

**Figure 1:**
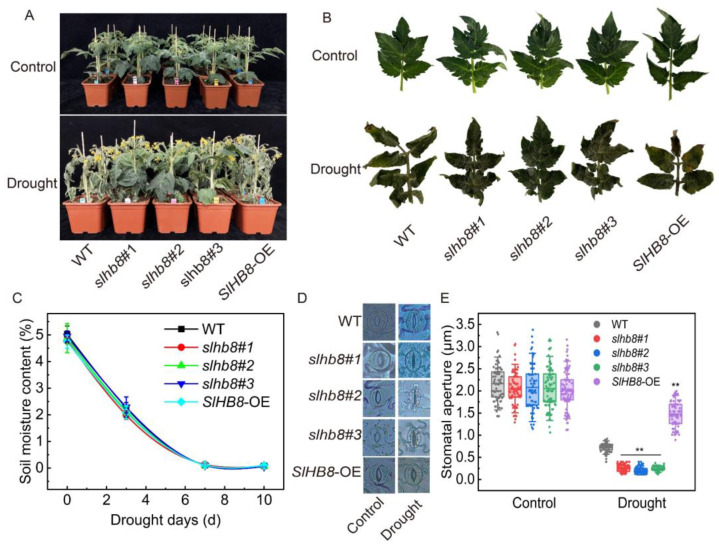
The effects of SlHB8 on leaf and stomata morphology of tomato plants subjected to drought stress. (**A**) Plant phenotypes; (**B**) detached leaf phenotypes; (**C**) soil percent moisture; (**D**) stomatal morphology; (**E**) stomatal aperture. Data are means ± standard deviation of four to six independent biological replicates. Significant differences in mean values are indicated by an asterisk: ** *p*-value < 0.01; Student’s *t*-test.

**Figure 2:**
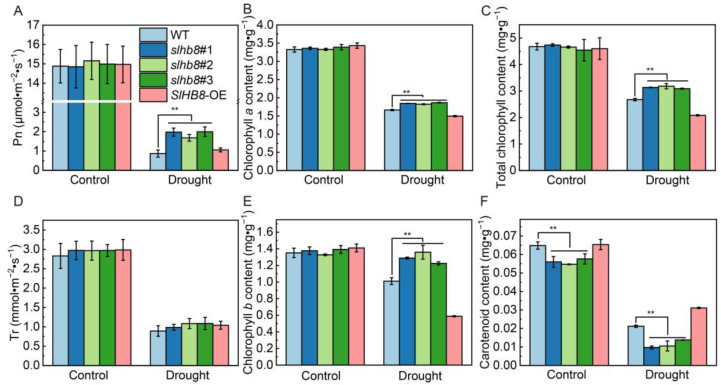
The effects of SlHB8 on photosynthetic rate and pigment content of tomato leaves under drought. (**A**) Net photosynthetic rate; (**B**) chlorophyll a content; (**C**) total chlorophyll content; (**D**) transpiration rate; (**E**) chlorophyll b content; (**F**) carotenoid content. Data are means ± standard deviation of four to six independent biological replicates. Significant differences in mean values are indicated by an asterisk: ** *p*-value < 0.01; Student’s *t*-test.

All the tomato plants of different genotypes grew well without drought treatment, except for the leaves of *SlHB8*-OE which exhibited an upward curled phenotype. After the drought treatment, the leaves of WT and *SlHB8*-OE showed severe wilting, while *slhb8* still maintained well-developed leaves (Figure 1A,B). The water content of the cultivated substrates decreased linearly within 7 days of treatment, reaching the lowest value and stabilizing thereafter; no significant difference was observed among the different materials (Figure 1C). Prior to drought treatment, the stomatal aperture of tomato leaves ranged between 2.0 and 2.5 μm, with no differences observed among the different materials. However, after the drought treatment, the stomatal width of all materials significantly decreased. Notably, statistical analysis revealed that the stomatal aperture of the three strains of *slhb8* was 32.26% of that of WT and 15.72% of that of *SlHB8*-OE (Figure 1D,E). Following drought stress, the net photosynthetic rate of *slhb8* was 50% higher than WT and *SlHB8*-OE, with no difference observed in transpiration rate (Figure 2A,D). Prior to drought treatment, there were no differences in chlorophyll a, chlorophyll b, and total chlorophyll content. However, the carotenoid content of *slhb8* was significantly lower than that of WT and *SlHB8*-OE. After drought treatment, the contents of chlorophyll and carotenoid significantly decreased, while the chlorophyll content in *slhb8* was significantly higher than that in WT and *SlHB8*-OE. Furthermore, the carotenoid content in *slhb8* was significantly lower than that in WT and *SlHB8*-OE (Figure 2B,C,E,F).

### 2.2. Effects of SlHB8 on Oxidative Stress and Antioxidant Enzyme Activity in Tomato Leaves under Drought Stress

**Figure 3:**
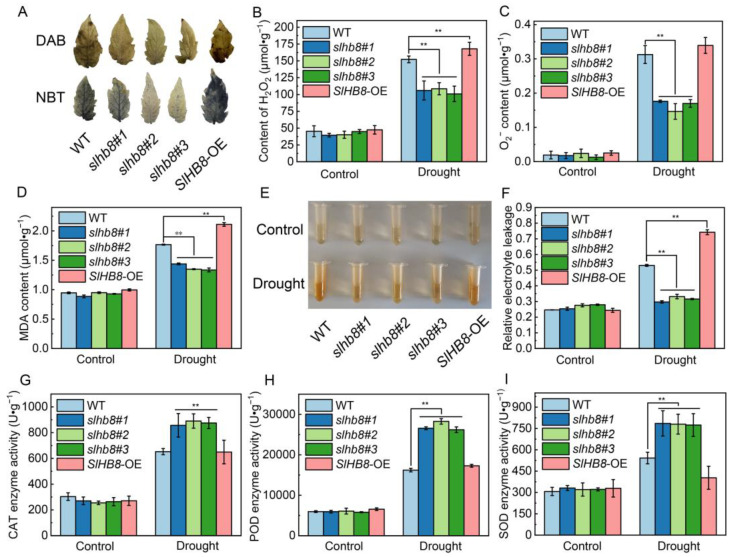
The effects of SlHB8 on oxidative stress and antioxidant enzyme activity in tomato leaves under drought treatment. DAB staining for detecting H_2_O_2_ production and NBT staining for indicating O_2_^−^ production (**A**); content of H_2_O_2_ (**B**); content of O_2_^−^ (**C**); content of MDA (**D**); the extract solution of MDA (**E**); relative electrolyte leakage (**F**); the activity of catalase (CAT) (**G**); the activity of peroxidase (POD) (**H**); the activity of superoxide dismutase (SOD) (**I**). Data are means ± standard deviation of four to six independent biological replicates. Significant differences in mean values are indicated by an asterisk: ** *p*-value < 0.01; Student’s *t*-test.

After the drought treatment, the leaves of WT and *SlHB8*-OE plants showed deeper nitro-blue tetrazolium chloride (NBT) and diaminobenzidine (DAB) staining, while the staining of *slhb8* plants was lighter (Figure 3A). The accumulation of H_2_O_2_ and O_2_^−^ was observed after drought treatment, being almost two-fold higher in WT and *SlHB8*-OE compared to *slhb8* (Figure 3B,C). The MDA extract solution showed lighter color rendering and lower MDA content without drought treatment, with no significant difference among different genotypes. However, after drought treatment, WT and *SlHB8*-OE plants exhibited deeper color rendering and significantly higher MDA content compared to *slhb8* (Figure 3D,E). The relative electrical conductivity of WT and *SlHB8*-OE leaves significantly increased after drought treatment, and was notably higher than that of *slhb8* plants (Figure 3F). These findings indicate that WT and *SlHB8*-OE cells experience more severe damage under drought stress. There was no difference in catalase (CAT), superoxide dismutase (SOD), and peroxidase (POD) activity among different genotypes without drought treatment. However, these enzyme activity of *slhb8* was significantly higher than that of WT and *SlHB8*-OE under drought treatment (Figure 3G–I).

### 2.3. Effects of SlHB8 on Photochemical Reaction Efficiency of Photosystems in Tomato Leaves under Drought

**Figure 4:**
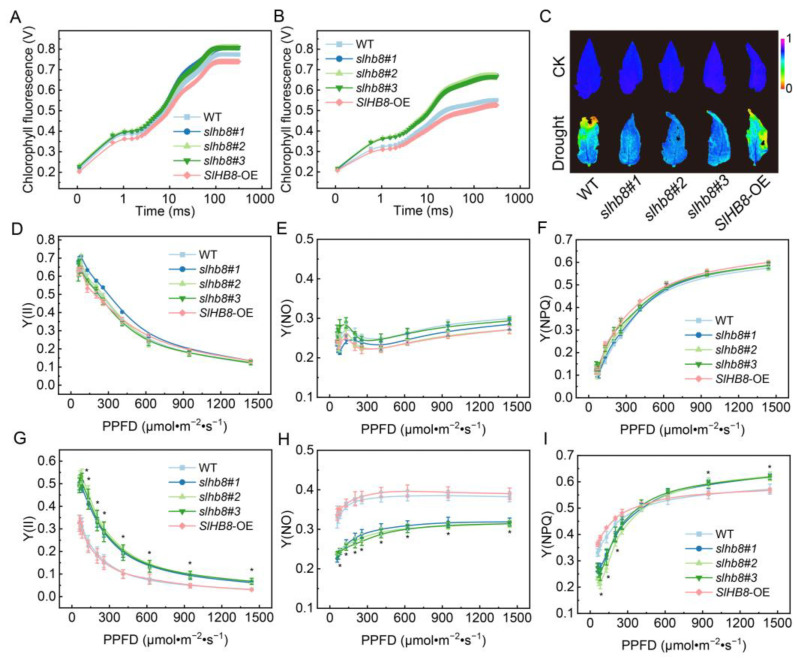
The effects of SlHB8 on photochemical reaction efficiency of photosystem II in tomato leaves under drought. Fast chlorophyll fluorescence signal curve in control group (**A**) and in drought treatment group (**B**); chlorophyll fluorescence imaging analysis of Fv/Fm (**C**); the effective photochemical quantum yield of PSII (Y(II)) in control group (**D**) and in drought treatment group (**G**); the quantum yield of non-regulated energy dissipation of PSII (Y(NO)) in control group (**E**) and in drought treatment group (**H**); the quantum yield of regulated energy dissipation of PSII (Y(NPQ)) in control group (**F**) and in drought treatment group (**I**). Data are means ± standard deviation of four to six independent biological replicates. Significant differences in mean values are indicated by an asterisk: * *p*-value < 0.05; Student’s *t*-test.

**Figure 5:**
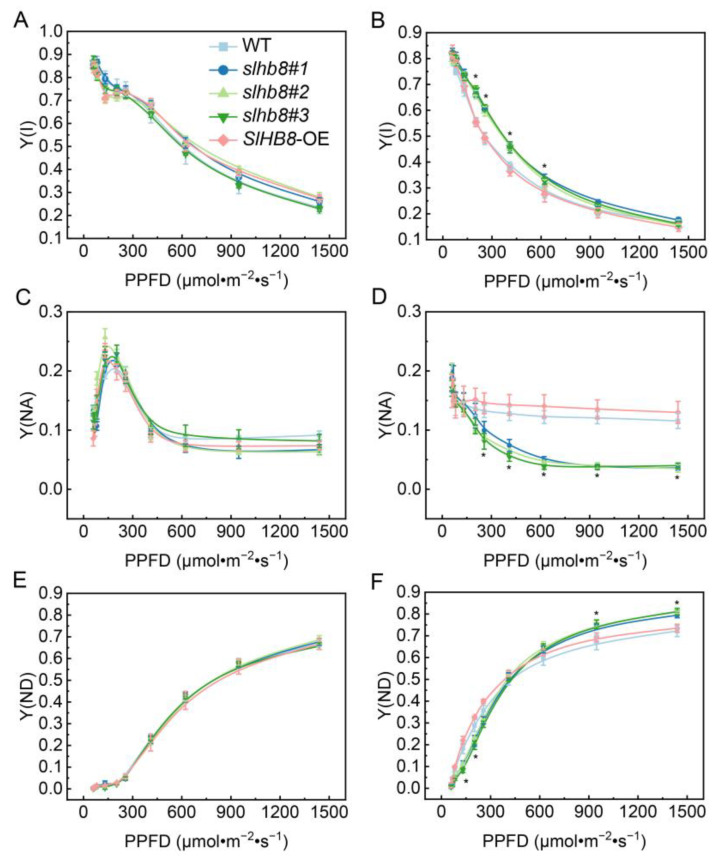
The effects of SlHB8 on photochemical reaction efficiency of photosystem I in tomato leaves under drought. The effective photochemical quantum yield of PSI (Y(I)) in control group (**A**) and drought treatment group (**B**); the quantum yield of non-photochemical energy dissipation due to acceptor-side limitation (Y(NA)) in control group (**C**) and in drought treatment group (**D**); the quantum yield of non-photochemical energy dissipation due to donor-side limitation (Y(ND)) in control group (**E**) and in drought treatment group (**F**). Data are means ± standard deviation of four to six independent biological replicates. Significant differences in mean values are indicated by an asterisk: * *p*-value < 0.05; Student’s *t*-test.

No significant difference was observed in the fast chlorophyll fluorescence induction kinetic curves of the leaves of different tomato materials without drought treatment. However, after drought treatment, the maximum signals of WT and *SlHB8*-OE were significantly weaker compared to *slhb8*. Similarly, there was no significant difference in the maximum photochemical reaction efficiency of PSII (Fv/Fm). After drought treatment, Fv/Fm decreased significantly, with *slhb8* demonstrating significantly higher values compared to WT and *SlHB8*-OE (Figure 4A–C). The results of the rapid light response curve showed no significant difference among Y(II), Y(NO), and Y(NPQ) without drought treatment (Figure 4D–F). After drought treatment, Y(II) showed a decreasing trend in all kinds of materials. Specifically, Y(II) in *slhb8* was significantly higher than in WT and *SlHB8*-OE, while Y(NO) was significantly lower. Y(NPQ) in *slhb8* was lower than in WT and *SlHB8*-OE under low light intensity, but higher under relatively high light intensity (Figure 4G–I). This suggests that *slhb8* can alleviate the adverse effects of drought stress on the activity of PSII and enhance the donor-side energy dissipation capacity of PSII under higher light intensity. In the absence of drought treatment, there was no significant difference in Y(I), Y(NA), and Y(ND) under different light intensities (Figure 5A,C,E). After drought treatment, Y(I) in *slhb8* was significantly higher than in WT and *SlHB8*-OE, and Y(NA) was significantly lower. Under low light intensity, Y(ND) in *slhb8* was lower than in WT and *SlHB8*-OE, but higher under strong light intensity (Figure 5B,D,F). This suggests that *slhb8* can alleviate the adverse effects of drought stress on the activity of PSI and enhance the donor-side energy dissipation capacity of PSI under higher light intensity.

### 2.4. Effects of SlHB8 on Chloroplast Ultrastructure in Tomato Leaves under Drought Stress

**Figure 6:**
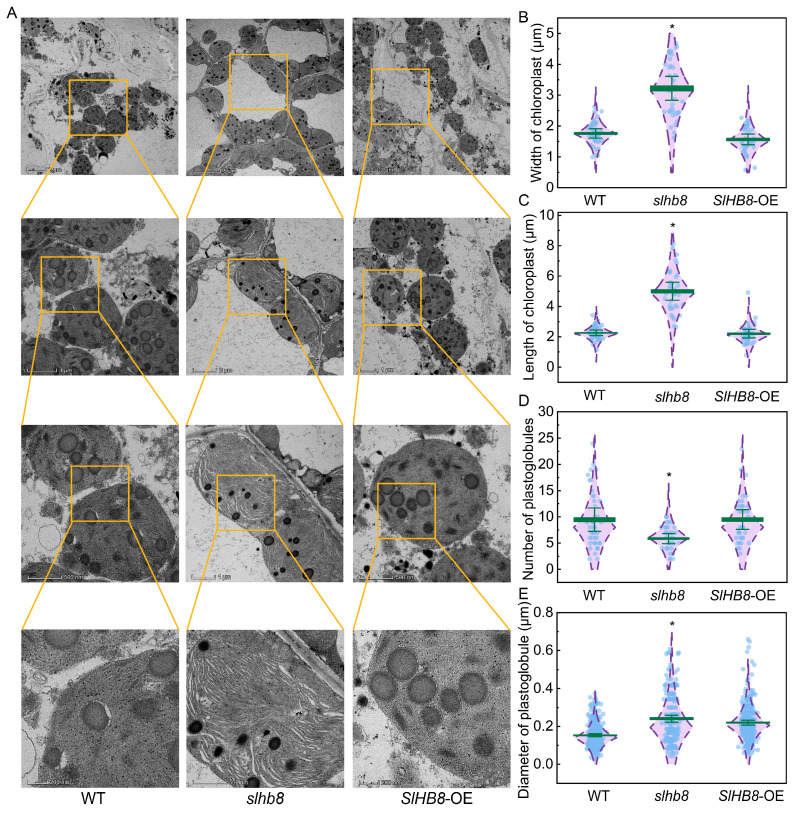
The effects of SlHB8 on chloroplast ultrastructure in tomato leaves under drought stress. chloroplast ultra-microstructure (**A**); width of chloroplast (**B**); length of chloroplast (**C**); number of plastoglobules (**D**); diameter of plastoglobules (**E**). Data are means ± standard deviation. Significant differences in mean values are indicated by an asterisk: * *p*-value < 0.05; Student’s *t*-test.

The chloroplast ultrastructure in tomato leaves was examined to assess the impact of SlHB8 during drought stress. The various genotype plants exhibited different levels of damage to chloroplast structures following drought treatment, resulting in irregular cell and chloroplast morphology, chloroplast degradation, severe damage to thylakoid crenellated structures, and the formation of numerous plastid glomeruli within the chloroplasts (Figure 6A). A statistical analysis was conducted to compare the differences in chloroplast morphology and plastoglobules among the different genotypes. The results demonstrated that the length and width of chloroplasts in the *slhb8* plants were nearly 100% higher compared to the WT and *SlHB8*-OE plants, resulting in a more complete chloroplast morphology and structure in *slhb8* plants. This indicated that *slhb8* mitigated the extent of chloroplast structure damage caused by drought stress in tomatoes (Figure 6B,C). Furthermore, the number of plastoglobules in WT and *SlHB8*-OE was 60% higher than *slhb8*, while the diameter of plastoglobules in WT and *SlHB8*-OE was significantly lower than *slhb8* (Figure 6D,E).

### 2.5. RNA-seq Analysis the Effects of SlHB8 on Gene Expression in Tomato Leaves under Drought Stress

**Figure 7:**
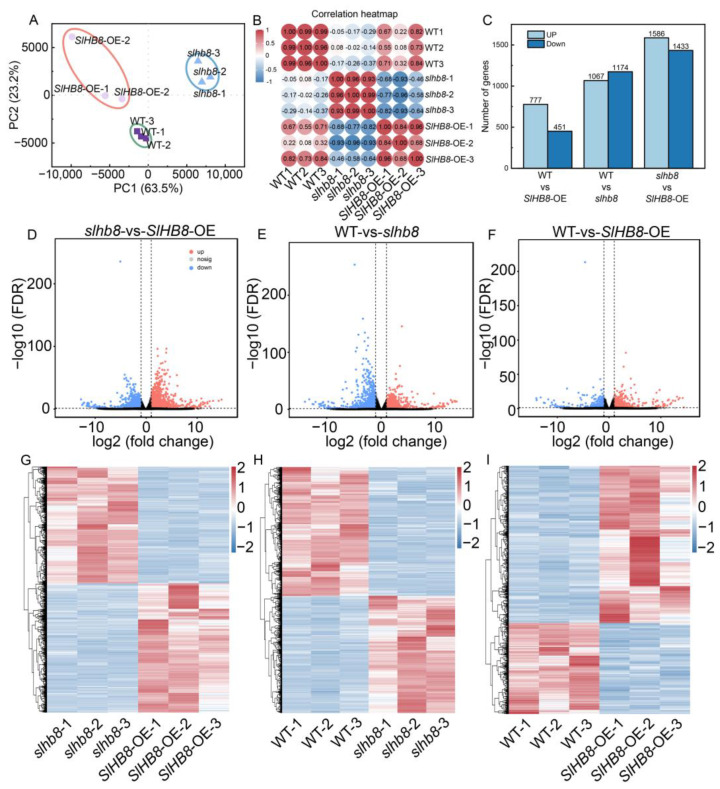
Analysis of differentially expressed genes (DEGs) of tomato leaves under drought stress. Principal component analysis (PCA) (**A**); heat diagram of correlation coefficient (**B**); the number of DEGs (**C**); volcano plot of DEGs between *slhb8* and *SlHB8*-OE (**D**); volcano plot of DEGs between WT and *slhb8* (**E**); volcano plot of DEGs between WT and *SlHB8*-OE (**F**); cluster heat map of DEGs between *slhb8* and *SlHB8*-OE (**G**); cluster heat map of DEGs between WT and *slhb8* (**H**); cluster heat map of DEGs between WT and *SlHB8*-OE (**I**).

**Figure 8:**
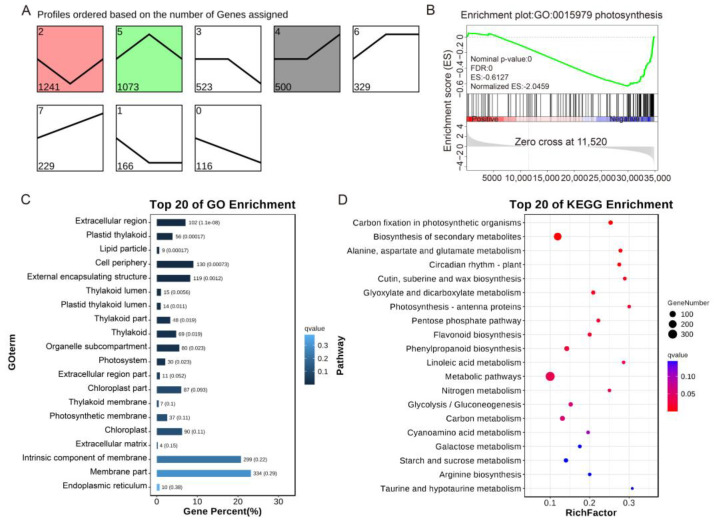
Differential expression gene enrichment analysis of SlHB8 regulation under drought. Profiles ordered based on the number of genes assigned (**A**); GSEA analysis of enrichment plot: GO0015979 photosynthesis (**B**); analysis of enriched GO terms (**C**) and KEGG metabolic pathways (**D**).

Principal component analysis (PCA) was performed on the RNA-seq data of three genotype plants, which revealed clear clustering of the three repeated samples from different groups and significant differences among the groups (Figure 7A). Correlation analysis further confirmed a high correlation between the three replicates of each sample (Figure 7B). Differential expression analysis showed that, compared to the WT, the overexpression of *SlHB* led to 777 upregulated genes and 451 downregulated genes. Similarly, loss of function of *SlHB8* resulted in 1067 upregulated genes and 1174 downregulated genes compared to WT. Furthermore, when comparing *slhb8* with *SlHB8*-OE, 1586 genes were upregulated and 1433 genes were downregulated (Figure 7C). Volcano plots and cluster heatmaps provided an overview of the differentially expressed genes in the various comparison groups (Figure 7D–I). To understand the expression trends of different genes across the materials, we identified eight distinct gene expression profiles. Profiles 2 and 5 exhibited significant trends with a *p*-value less than 0.01, consisting of 1241 and 1073 genes, respectively. These profiles indicated that the gene expression difference between WT and *SlHB8*-OE was minimal, while the difference between both of them and *slhb8* was substantial (Figure 8A). Given that there were no apparent phenotypic or sequencing differences between WT and *SlHB8*-OE after drought treatment, but both were significantly different from *slhb8*, we focused our analysis on the possible biological pathways associated with the differentially expressed genes in the *slhb8* and *SlHB8*-OE comparison group. Gene set enrichment analysis (GSEA) of this group revealed that *SlHB8*-OE downregulated the expression of most photosynthesis-related genes compared to *slhb8* (Figure 8B). GO analysis of the *slhb8* and *SlHB8*-OE comparison group identified the top 20 enriched GO terms, which mainly included thylakoids, chloroplasts, and photosystems, all of which are closely related to leaf photosynthesis (Figure 8C). Moreover, the enrichment of KEGG metabolic pathways highlighted carbon dioxide fixation, photosynthetic antenna pigment, starch and sugar metabolism, and flavonoid metabolism (Figure 8D). These findings suggest that the knockout of *SlHB8* affects the expression of a significant number of genes related to photosynthesis in response to drought stress.

### 2.6. Analysis of Gene Expression Related to Photosynthesis and Drought Resistance

**Figure 9:**
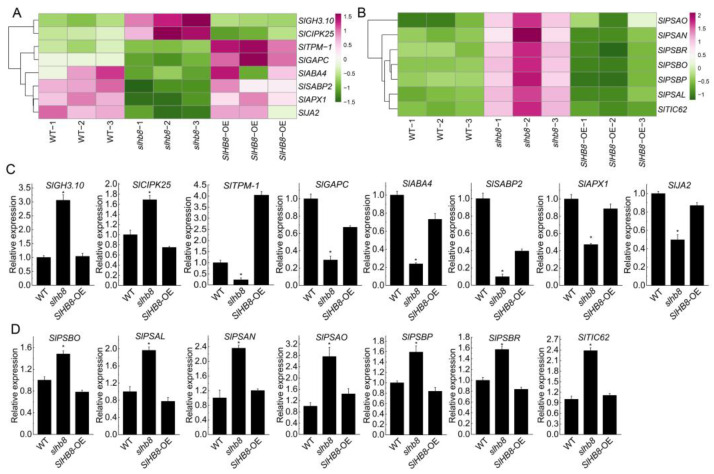
Analysis of genes expression related to photosynthesis and stress resistance signal regulated by SlHB8 under drought stress. The expression of selected stress signal-related genes in the transcriptome sequencing results (**A**); the expression of selected photosynthesis-related genes in the transcriptome sequencing results (**B**); qRT-PCR validates the expression of stress signal-related genes (**C**); qRT-PCR validates the expression of photosynthesis-related genes (**D**). Data are means ± standard deviation of three independent biological replicates. Significant differences in mean values are indicated by an asterisk: * *p*-value < 0.05; Student’s *t*-test.

We conducted an analysis of differentially expressed genes associated with drought resistance using transcriptome sequencing data. Among these genes, we observed a significant upregulation of *SlGH3.10* and *SlCIPK25* in *slhb8* tomato plants. Conversely, *SlTPM-1*, *SlGAPC*, *SlABA4*, *SlSABP2*, *SlAPX1*, and *SlJA2* were downregulated. The expression patterns of these genes were consistent with the results obtained from qRT-PCR analysis (Figure 9A,C). Additionally, several key genes related to photosynthesis, including *SlPSAO*, *SlPSAN*, *SlPSAL*, *SlPSBO*, *SlPSBP*, *SlPSBR*, and *SlTIC62*, were significantly upregulated in *slhb8* tomato plants. The expression trends of these genes were consistent with the qRT-PCR results (Figure 9B,D). These findings suggest that the expression of genes associated with jasmonic acid, salicylic acid, abscisic acid, calcium, and photosynthesis play a role in the regulation of drought resistance by the transcription factor SlHB8.

### 2.7. Analysis of Water Absorption Capacity of Plants and Expression Analysis of Aquaporin Genes

**Figure 10:**
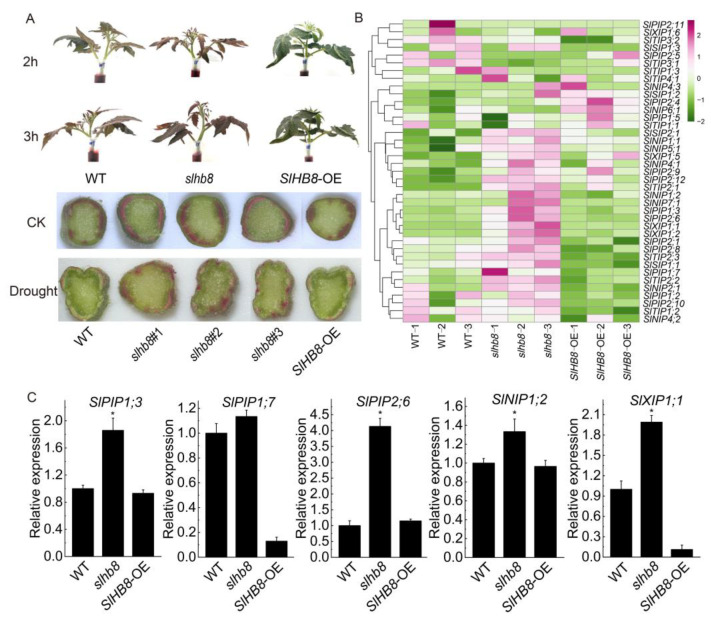
The evaluation of water absorption capacity and expression analysis of the aquaporin-related genes regulated by SlHB8 under drought stress. Water absorption capacity test of tomato plants by using red ink (**A**); analysis of the gene expression of the aquaporin protein family based on the transcriptome sequencing results (**B**); qRT-PCR validation of the expression pattern of aquaporin-related genes (**C**). Data are means ± standard deviation of three independent biological replicates. Significant differences in mean values are indicated by an asterisk: * *p*-value < 0.05; Student’s *t*-test.

A water absorption capacity test was conducted on tomato plants using red ink. After 2 and 3 h of water absorption, it was observed that the entire plant of *slhb8* exhibited a distinct red color, while the red color in *SlHB8*-OE plants was very light. This observation is consistent with the results obtained from the examination of stem cross-sections (Figure 10A). The transport of water is closely associated with aquaporin proteins; the gene expression of the aquaporin family was analyzed in the transcriptome sequencing results, revealing that more than half of the aquaporin genes were upregulated in the *slhb8* plants. Furthermore, qRT-PCR verification results indicated that the expression of *SlPIP1;3*, *SlPIP2;6*, *SlTIP3;1*, *SlNIP1;2*, and *SlXIP1;1* was upregulated in the *slhb8* plants, which is consistent with the transcriptome sequencing results (Figure 10B,C). These pronounced differences in results suggest that aquaporins may play a role in the water absorption of plants, which could be related to SlHB8-regulated drought resistance.

## 3. Discussion

### 3.1. The Role of HD-ZIP III Proteins in Plant Development and Stress Resistance

HD-ZIP III proteins have been widely identified to be involved in the regulation of plant development. For example, HD-ZIP III genes were found to be upregulated during adventitious bud regeneration in apple plants [22]. During the root development of *Arabidopsis*, HD-ZIP III transcription factors play a crucial role in controlling the periclinal division of vascular cells, which involves the expressional regulation of genes related to brassinosteroid biosynthesis [23]. Furthermore, HD-ZIP III transcription factors have also been implicated in leaf senescence by directly regulating the expression of senescence-associated genes [24]. In previous studies, we demonstrated that the overexpression of *SlHB8* resulted in decreased lignin content and downregulation of lignin biosynthesis-associated genes in both leaves and stems, leading to an upward leaf rolling phenotype [13,14]. Additionally, our observations on pollen development revealed that *SlHB8* overexpression in tomato plants reduced pollen activity and subsequently inhibited fruit setting, while the CRISPR/Cas9-mediated *slhb8* mutant displayed opposite phenotypes [15]. In this study, we further investigated the role of SlHB8 in regulating abiotic stress resistance by utilizing available overexpression and gene editing materials. Our findings showed that *slhb8* increased drought resistance, particularly by alleviating oxidative damage and photosynthetic inhibition caused by drought stress. It is worth noting that HD-ZIP family transcription factors also play a regulatory role in the abiotic stress response in plants [19,25]. For instance, cold and salt stress can induce the expression of most HD-ZIP genes in watermelon [26]. Similarly, several *ZmHDZ* genes were found to be upregulated under drought stress and their promoter regions contain several regulatory elements associated with drought tolerance, suggesting that *ZmHDZ* genes may be involved in drought tolerance in maize [27]. Moreover, HD-ZIP III proteins have been identified as targets of a conserved stress biomarker microRNA 166, forming a regulatory module that fine-tunes the response to stressful environments [16].

### 3.2. The Loss of Function of SlHB8 Can Effectively Alleviate Oxidative Damage Caused by Drought Stress in Tomato Plants

In this study, we observed that the *slhb8* mutant exhibited stronger phenotypes compared to the WT and *SlHB8*-OE plants under drought stress (Figure 1A,B). Despite the significant accumulation of ROS in the leaves caused by drought stress, it is evident that *slhb8* mitigated the accumulation of H_2_O_2_ and O_2_^−^, which corresponded with the levels of MDA and relative electrolyte leakage in the leaves (Figure 3D–F). These findings indicate that the transcription factor SlHB8 negatively regulates oxidative toxicity in tomato leaves induced by drought stress. It is known that external environmental stimuli, such as drought, can disrupt the steady-state level of ROS, leading to intracellular oxidative damage [28]. Plants possess defense mechanisms, including enzymatic and non-enzymatic pathways mediated by plasma membrane-localized respiratory burst oxidase homologs (RBOHs), to regulate ROS production and scavenging, effectively maintaining the balance of intracellular ROS and alleviating stress-induced damage [29]. A previous study in apple trees demonstrated that the HD-Zip I transcription factor MdHB7 positively regulates drought tolerance by increasing the activities of POD and SOD to reduce ROS accumulation [30]. It is worth noting that the discrepancy between our results and the findings regarding MsHB7 may be attributed to the fact that HB7 and HB8 belong to different subfamilies. In our study, we found that the reduction in ROS accumulation in tomato leaves under drought stress in the *slhb8* mutant could be attributed to the increased activity of antioxidant enzymes (Figure 3G–I).

### 3.3. The Role of SlHB8 in Mediating Drought Resistance Involves the Regulation of Photosynthesis and the Maintenance of Chloroplast Quality

In this study, we observed that drought stress significantly led to stomatal closure, with the *slhb8* mutant showing more pronounced effects compared to the WT and *SlHB8*-OE plants, resulting in reduced water loss (Figure 1D,E). In addition to stomatal dynamics, the activity of photosystems in tomato plants is highly sensitive to environmental fluctuations, including low temperature, salinity, and drought. Various mechanisms have been reported in previous studies to adapt to these fluctuations [6,11,31]. Consistent with recent research utilizing biochemical and spectroscopic methods to investigate the response of the photosynthetic apparatus to drought stress, we found that drought stress significantly inhibited the photochemical reaction efficiency of both PSII and PSI [32]. Remarkably, the knockout of *SlHB8* in plants effectively mitigated the degree of photoinhibition of the photosystems caused by drought stress, and maintained higher values of Y(NPQ) and Y(ND) under relatively high light intensity (Figure 4G–I and Figure 5D–F). NPQ is a crucial short-term regulatory mechanism involved in balancing light-dependent photosynthetic processes [33]. In our study, the elevated Y(ND) and reduced Y(NA) indicate an enhanced ability of *slhb8* plants to recycle electrons from the PSI acceptor side to the donor side for the oxidation of P700 (Figure 5D,F). The oxidation of P700 serves as a robust photoprotective mechanism, alleviating PSI photoinhibition [34].

Drought stress can induce severe photoinhibition of the photosystem, leading to photodamage and degradation of chloroplasts. Our results from transmission electron microscopy clearly demonstrated that drought has caused significant damage to the morphology and structure of chloroplasts. However, the *slhb8* mutant showed a notable alleviation of chloroplast damage caused by drought (Figure 6A). Proteins within the photosynthetic apparatus are highly vulnerable to drought stress, as toxic ROS are produced at both photosystems during the photochemical reaction. This highlights the significance of the photosynthetic electron transport chain at the thylakoid membranes as a target for regulating chloroplast quality [35]. We observed a substantial accumulation of plastoglobules (PGs) in the chloroplasts, particularly in the WT and *SlHB8*-OE plants, indicating severe damage to the chloroplast structure (Figure 6D,E). PGs play important roles in carotenoid metabolism, redox and photosynthetic regulation, prenyl lipids, plastid biogenesis, and senescence, including the recycling of thylakoid lipid components [36,37]. The size and shape of PGs are dynamically regulated by approximately 30 core proteins in response to abiotic stress. The accumulation of PGs under stress conditions is closely associated with chloroplast structural damage caused by photoinhibition, thereby contributing to environmental adaptation. Our findings suggest that the knockout of *SlHB8* can reduce the extent of drought-induced damage to chloroplasts.

### 3.4. Expression of Drought-Related Genes and Water Absorption Capacity Regulated by SlHB8 in Drought Resistance

Plants have developed multiple mechanisms to cope with stress conditions during their growth. Drought stress triggers a signaling cascade that induces short-term stomatal responses to reduce water loss, as well as long-term responses involving physiological and morphological changes through the remodeling of the transcriptional network [38]. In our previous RNA-seq study, we discovered that SlHB8 regulates the expression of photosynthesis-antenna proteins, genes associated with response to external stimuli and stress, and genes involved in pollen wall development in tomato plants [13,14,15]. However, the transcriptional regulation of SlHB8 under drought conditions remains unclear. In this study, our RNA-seq results revealed that the knockout of *SlHB8* induced the expression of a significant number of genes associated with metabolism and chloroplast integrity. These genes play a crucial role in the regulation of drought resistance in tomato plants by SlHB8. Over the past few decades, the positive effects of the ABA-associated signaling pathway and several master transcriptional regulatory factors on enhancing plant drought resistance have been identified [39]. In our present study, we also found that SlHB8 regulates genes involved in photosynthesis-antenna proteins, ABA signaling, and other drought-related processes in response to drought stress.

In addition, the water absorption capacity of plants is a crucial aspect of plant drought resistance and the study of the aquaporin transport system mediating drought stress in plants has garnered increasing attention in recent years [40,41,42]. Genome-wide association analysis and genetic research have identified an aquaporin gene *PvXIP1;2* that confers drought resistance in common beans [43]. The rice aquaporin *OsPIP2;2* efficiently facilitates the transport of H_2_O to protect rice cells from damage caused by drought stress [44]. In *Arabidopsis*, the aquaporin PIP2;8 has been identified; it can be directly regulated by A/T-rich protein and zinc-binding protein transcription factors PLATZ4, positively influencing plant drought tolerance [45]. In this study, we also found that SlHB8 is capable of negatively regulating the water absorption capacity of tomato plants and the expression of certain aquaporin genes. These findings suggest that SlHB8 may play a role in regulating the drought resistance of tomatoes. However, in-depth molecular experiments are needed to verify the target genes directly regulated by SlHB8 and to elucidate the potential biological processes, such as photoprotection and water absorption capacity in plants, that are involved in the mechanism of SlHB8 in regulating drought resistance.

## 4. Materials and Methods

### 4.1. Plant Materials and Treatments

The wild-type (WT) tomato cultivar ‘Micro-Tom’ (*Solanum lycopersicum* L.) was used as the control; in addition, homozygous *SlHB8*-overexpression (*SlHB8*-OE) and knockout mutants generated through CRISPR/Cas9 technology (*slhb8*) from tomato plants previously obtained were utilized in this study [13,14,15]. The plants were cultivated in an artificial climate room at the South China Agricultural University, with a temperature of approximately 25 °C during the day and 15 °C at night. Additionally, an artificial light source was provided, with a light intensity of approximately 300 μmol·m^−2^·s^−1^. The light–dark cycle was set to 16 h of light and 8 h of darkness. The tomato plants were grown in 10 × 10 cm pots filled with a mixture of imported peat, vermiculite, and perlite with volume ratio 2:1:1. Normal irrigation was conducted. The WT, *SlHB8*-OE, and *slhb8* plants with uniform growth and five leaves were selected for the treatments. Prior to the treatments, all plants received a standardized amount of irrigation. The drought resistant examination experiment consisted of a control group with normal irrigation and a drought treatment group with no irrigation. After 9 days of water deficit treatment, leaf samples from each strain were collected to determine the physiological response. The weight method was employed to calculate the soil percent moisture content based on the mass of the soil before and after drying.

### 4.2. Observation of Stomatal Aperture

At least six replicates of leaf samples from each treatment group were randomly collected. The abaxial lower epidermis of the leaves was carefully removed using tweezers and incubated in distilled water on a new microscope slide. The stomatal aperture of tomato leaves was observed and photographed using an inverted fluorescence microscope (Zeiss, Oberkochen, Germany). Approximately 10 random microscopic fields were taken for each leaf and the parameters of stomatal aperture were measured using Image J software (version number: 1.8.0).

### 4.3. Determination of Photosynthetic Pigment Content

An amount of 0.1 g fresh leaf samples, with the center veins removed, were immersed in a 15 mL test tube containing 10 mL of an extracting solution consisting of 95% ethanol. The samples were soaked in darkness for 24 h. The optical density (OD) values at 663 nm, 646 nm, and 470 nm were measured using a UV 1200 ultraviolet spectrophotometer (Shimadzu, Kyoto, Japan). The photosynthetic pigment content was then calculated according to the methods previously described [46].

### 4.4. Determination of Oxidative Stress and Antioxidant Enzyme Activity

The fresh leaves were carefully put into a 50 mL centrifuge tube containing staining fluid, either DAB or NBT, and incubated overnight at room temperature in the dark. Once the leaves were adequately stained, the leaf pigments were removed by immersing them in anhydrous ethanol. The fully bleached leaves were then observed.

The content of malondialdehyde (MDA) in tomato leaves was determined using thiobarbituric acid (TBA). Leaf samples weighing 0.2 g were ground until homogenized and then immersed in 2 mL of 10% trichloroacetic acid (TCA). The mixture was centrifuged at 4000 rpm for 30 min. Afterwards, 1 mL of the collected supernatant was mixed with 1 mL of 0.6% TBA solution and subjected to a 15 min boiling water bath. The reaction was immediately terminated by placing the mixture in an ice bath. The absorbance was measured to calculate the optical density value.

The relative electrolyte leakage was determined as previously described. Briefly, six leaf disks were sampled from the leaves and placed in tubes with 10 mL of deionized water at room temperature for 12 h. The conductivity was measured using a conductivity meter before and after the tubes were bathed in boiling water for 30 min.

To further quantitatively analyze ROS accumulation in tomato leaves, the O_2_^−^ production rate and H_2_O_2_ content were determined using commercial enzyme-linked immunosorbent assay (ELISA) kits (Solarbio, Beijing, China). Additionally, the activity of the endogenous superoxide dismutase (SOD), catalase (CAT), and peroxidase (POD) was measured using commercial ELISA kits (Solarbio, Beijing, China). Sample preparation and testing procedures were strictly carried out in accordance with the manufacturer’s instructions.

### 4.5. Measurement of Photosynthetic Gas Exchange Parameters

In this study, the GFS-3000 photosynthesizer connected with the Dual-PAM-100 chlorophyll fluorescence analyzer (Heinz Walz, Effeltrich, Germany) was used to measure the carbon dioxide fixation rate (Pn) and transpiration rate (E). Carbon dioxide in the air was used as a reference. Illumination of the leaf was provided by the Dual-PAM-100 with an intensity of 1100 μmol·photos·m^−2^·s^−1^ of artificial light. The parameters were recorded once the leaf photosynthesis reached a stable state.

### 4.6. Measurement of Photosystem Activity

Prior to the chlorophyll fluorescence measurement, all plants were acclimated in complete darkness for 30 min. The fast chlorophyll fluorescence induction kinetics curve was measured using a saturated pulse light with an intensity of 10,000 μmol·m^−2^·s^−1^ and a duration of 300 ms. Subsequently, the slow chlorophyll fluorescence induction kinetics were recorded with a light intensity of 214 μmol·m^−2^·s^−1^ for a duration of 6 min, following our previously described method with minor modifications [11]. Additionally, the rapid light response curves (RLCs) were measured using varying light intensities of 59, 68, 81, 131, 202, 256, 410, 622, 947, and 1439 μmol·photons·m^−2^·s^−1^. The light intensity was increased every 30 s.

### 4.7. Observation of Chloroplast Structure by Transmission Electron Microscopy

First, veinless strips from fresh tomato leaves were fixed using a solution of 1% acetic acid and 2.5% glutaraldehyde. The samples were then dehydrated using a gradient of ethanol concentrations. After that, the samples were embedded in epoxy resin, sliced, stained, and cut into ultra-thin slices using an ultra-thin slicing machine (Leica EM UC7, Wetzlar, Germany). Finally, the transmission electron microscope (Hitachi HT-7700, Tokyo, Japan) was used to observe and photograph the ultra-microstructure of the chloroplasts in tomato leaves.

### 4.8. Transcriptomic Analysis

Tomato leaves were frozen and ground using liquid nitrogen. Total RNA was extracted using the TRIzol reagent kits according to the manufacturer’s instructions (Invitrogen, Waltham, MA, USA). The sequencing was performed using the Illumina Novaseq 6000 sequencing system by Gene Denovo Biotechnology Co. (Guangzhou, China). To quantify mRNA expression abundance and variations, the fragment per kilobase of transcript per million mapped reads (FPKM) value was calculated. Differentially expressed genes (DEGs) between two different groups were identified based on the following criteria: an absolute fold change of ≥2 and a false discovery rate (FDR) below 0.05. Furthermore, analyses were conducted using Gene Ontology (GO) annotation and Kyoto Encyclopedia of Genes and Genomes (KEGG) pathways to assess the DEGs between the two groups. Gene set enrichment analysis (GSEA) was performed to determine whether a set of genes in specific GO terms or KEGG pathways exhibited significant differences between the two groups. Additionally, gene expression pattern analysis was utilized to cluster genes with similar expression patterns across multiple samples. Advanced heatmaps were generated using OmicStudio tools available at https://www.omicstudio.cn (accessed on 8 June 2023). Each treatment consisted of three biological replicates.

### 4.9. Quantitative Real-Time Polymerase Chain Reaction Assays

Total RNA was extracted from 0.2 g of leaves using the Trizol reagent kit (Invitrogen, Carlsbad, CA, USA). Subsequently, 1 μg of RNA was reverse-transcribed into cDNA using the Prime Script RT Master Mix (Perfect Real Time, Takara, Kusatsu, Japan). Quantitative real-time polymerase chain reaction (qRT-PCR) was performed using the Super Real Pre-Mix Plus (SYBR Green) (TaKaRa, Dalian, China) and amplification was conducted on the 7500 Real-Time PCR System (Applied Biosystems, Foster City, CA, USA). The *Actin* housekeeping gene was used for gene expression analysis. The relative expression of genes was determined using the 2^−ΔΔCT^ method [13,14,15]. The primers used in this study are listed in Table S1.

### 4.10. Assessment of Red Ink Absorption Capacity of Tomato Plants

To assess the red ink absorption capacity of tomato plants, the roots of the tomato seedlings were first cut off. Next, the stem was inserted into a 15 mL centrifuge tube and the incision in the stem was submerged in red ink. After 2 h and 3 h of red ink absorption, the plants were photographed using a camera. Additionally, the cross-section of the stem was observed using a stereomicroscope (Olympus DP71, Tokyo, Japan).

### 4.11. Statistical Analysis

Statistical analysis and figure plotting were performed using SPSS version 22 (SPSS, Armonk, NY, USA) and Origin Version 12.0 (Systat, San Jose, CA, USA). Student’s *t*-tests were conducted to assess the significance of differences between treatments, with a significance threshold set at *p* ≤ 0.05.

## 5. Conclusions

In conclusion, *slhb8* mutant plants exhibited a robust resistant phenotype under drought stress, effectively mitigating oxidative damage, photoinhibition, and chloroplast damage induced by drought stress. The regulation of water absorption capacity and the expression of drought resistance-related genes such as *SlCIPK25*, *SlABA4*, and *SlJA2* were involved in this process. We conclude that SlHB8 plays a negative regulatory role in the drought resistance of tomato plants. These findings expand our understanding of the role of HD-ZIP transcription factors in regulating abiotic stress resistance and offer new insights for enhancing drought tolerance in plants. Additionally, the reliable data on photosynthetic physiology and transcriptome sequencing can serve as a valuable reference for future research.

## Data Availability

The data that supporting the findings are included in the article, the raw data of transcriptome sequencing this study are openly available in the National Center for Biotechnology Information (NCBI) SRA database under the BioProject ID: PRJNA1013493.

## Supplementary Materials

The supporting information can be downloaded at: https://www.mdpi.com/article/10.3390/ijms242015498/s1.
